# Treatment of Monteggia-like-lesion in a young patient: A case report

**DOI:** 10.1097/MD.0000000000024928

**Published:** 2021-05-14

**Authors:** Agung R. B. Santoso, Thomas E. C. J. Huwae, Anindita E. P. Wijaya, Alva Pribadi, Felix Cendikiawan, Muhammad Abduh

**Affiliations:** Orthopaedic and Traumatology Department, Faculty of Medicine Universitas Brawijaya, Saiful Anwar General Hospital, Malang, Indonesia.

**Keywords:** internal fixation, Monteggia-like-lesions, radial head fracture

## Abstract

**Introduction:**

A Monteggia fracture was described initially as a fracture of the proximal third ulna and anterior dislocation of the proximal epiphysis radius.^[1]^ In 1967, Bado discovered “true Monteggia lesions” and classified them into 4 groups.^[2]^ He also used the term “equivalents” or “Monteggia-like-lesions” to describe specific injuries with similar radiographic patterns.^[3]^ This type of fracture is rare and frequently associated with complications, poor functional results, and further operations.^[4]^

**Patient concerns:**

A 16-year-old girl was admitted to our emergency department after a single motorcycle accident. Her main complaint was the pain and swollen of her left elbow. She was reluctant to move her arm due to pain.

**Diagnosis:**

Radiograph examination showed a displaced fracture of the left proximal third ulna accompanied by displacement of the left proximal radius. This fracture was similar to the Monteggia type III fracture except for proximal radial disruption that occurred laterally through a Salter-Harris type II fracture.

**Interventions:**

The patient underwent surgical debridement, and the forearm was immobilized using a backslap in a supine position and elbow flexion 90^o^. Open reduction and internal fixation were performed 5 days later. The ulna was reduced and stabilized first using a 3.5 mm one-third tubular plate (ORMED), and internal fixation of the radial epiphysis was done using a 1.6 mm miniplate (Prohealth).

**Outcomes:**

After 3 months, the patient showed improvement with the Mayo Elbow Performance Score (MEPS) of 85. She did not complain of any pain and decreased strength. The patient regained 0 to 125^o^ of elbow flexion and 0 to 165^o^ of supination and pronation.

**Conclusion:**

Monteggia-like-lesion has many variations in physical and radiograph appearance. Careful evaluation of fracture pattern, identification of injury mechanism, and appropriate treatment planning based on Monteggia fracture treatment principles are mandatory to achieve the patient's best outcome.

## 1 Introduction

A Monteggia fracture was described initially by Giovanni Battista Monteggia in 1814, with the original description is a traumatic lesion distinguished by a fracture of the proximal third ulna and anterior dislocation of the proximal epiphysis radius.^[1]^ The Monteggia fracture can be considered a rare injury with less than 1% incidence of all fractures and 2% to 5% of all proximal forearm fractures.^[5]^ Unfortunately, this type of fracture-dislocation is frequently associated with complications, poor functional results, and further operations.^[4]^

**Figure 1:**
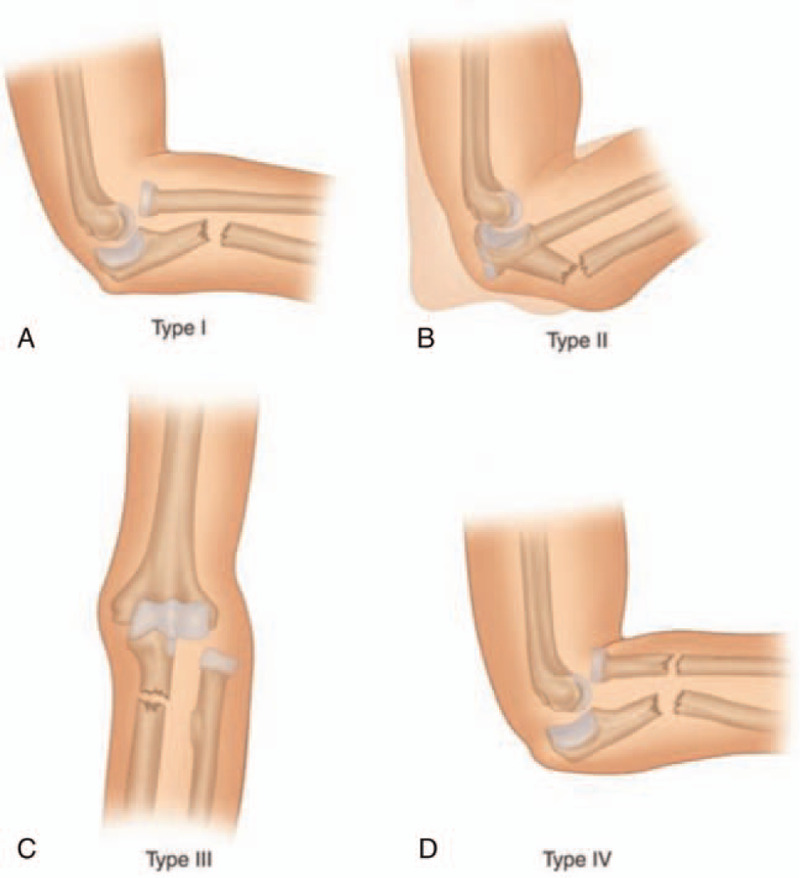
Bado's classification of Monteggia fractures (A) fracture ulnar diaphysis with anterior angulation and anterior dislocation radial head, (B) fracture ulnar diaphysis with posterior angulation and posterior dislocation radial head, (C) fracture ulnar metaphysis with lateral or anterolateral dislocation radial head, (D) fracture forearm with anterior dislocation of the radial head.

In 1967, Bado discovered “true Monteggia lesions” and classified them into 4 groups based on the direction of the radial head dislocation and the angulation of the ulna fracture.^[6]^ Type I fracture is a fracture of the ulnar diaphysis with anterior angulation of the bone stump, associated with the radial head's anterior dislocation. The injury mechanism is a direct force from the dorsal side to the proximal ulna or an indirect force due to a fall on the outstretched hand. The incidence of this type is about 15% of all Monteggia fracture. Type II fracture is a fracture of the ulnar diaphysis with posterior angulation of the bone stump, associated with the radial head's posterior dislocation. The mechanism of injury is an axial force to the 90^o^ bent elbow. This type is the most common Monteggia fracture, with an incidence is about 80% of all Monteggia fractures. Type III fracture is a fracture of the ulnar metaphysis with lateral or anterolateral dislocation of the radial head. The mechanism of injury is an abduction force with simultaneous pronation or supination. Type IV fracture is a fracture of the forearm with anterior dislocation of the radial head. The exact mechanism of injury is unknown but assumed to be similar to the type I fracture accompanied by a radius fracture. Type III and IV fractures are rare, with a total incidence is about 5%.^[5]^ Bado's classification of Monteggia fractures is seen in Figure 1.^[2]^

**Figure 2:**
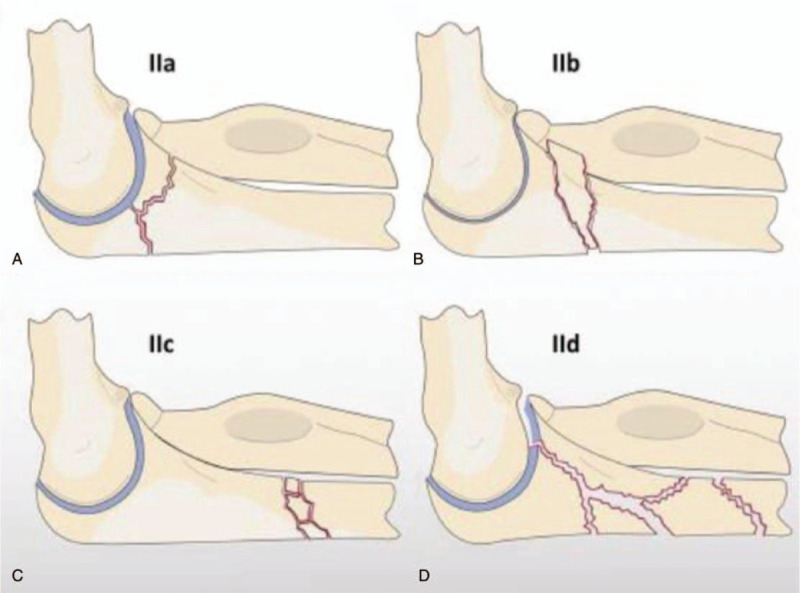
Jupiter's classification of Monteggia type II fractures (A) the fracture of ulna involves distal part of the olecranon and coronoid process, (B) the fracture is at metaphyseal-diaphyseal juncture distal to coronoid process, (C) the fracture is at diaphyseal ulna, (D) the fracture extends to the proximal half ulna.

The type II fracture has been subdivided into 4 groups by Jupiter et al In type IIA, the ulna's fracture involves the distal part of the olecranon and the coronoid process. In type IIB, the fracture is at the metaphyseal-diaphyseal juncture distal to the coronoid process. In type IIC, the fracture is at the diaphyseal ulna. Whereas in type IID, the fracture extends to the proximal half of the ulna.^[4]^ Jupiter's classification of Monteggia type II fractures is seen in Figure 2.^[7]^

Bado also used the term “equivalents” or “Monteggia-like-lesions” to describe specific injuries with different radiological appearances but possessed similar characteristics, especially in the mechanism of injury and its treatment.^[3]^ There are 4 types of Monteggia equivalents that contain pattern differences from each other (Table 1).^[8]^

**Table 1:** Various pattern of Monteggia equivalents (Gundavda and Chinoy, 2015)^[8]^.

Type	Patterns
Type I Monteggia equivalents	Isolated anterior dislocation of radial head (with plastic deformation of ulna)
	Isolated radial neck fracture
	Pulled elbow syndrome
	Fracture of the ulnar diaphysis with fracture of radial neck
	Fractures of both bones in forearm (wherein, the radial fracture is above the junction of the proximal and the middle third)
	Fracture of ulnar diaphysis with anterior dislocation of radial head and an olecranon fracture
	Fracture of ulnar diaphysis (at proximal and middle third junction) with displaced extension type supracondylar fracture of humerus (present case)
Type II Monteggia equivalents	Posterior elbow dislocation in children
Type III Monteggia equivalents	Displaced fracture of the lateral condyle of humerus
Type IV Monteggia equivalents	Distal humerus fracture with proximal third ulnar diaphysis fracture and distal radial metaphyseal fracture with anterior dislocation of radial head

Basic principles of treatment are early detection of the type of fracture, open reduction, stable internal fixation of the ulna, mostly open reduction of the radial head, and short immobilization time.^[5]^ The outcomes of operative treatment in Monteggia fracture-dislocations have been historically unpredictable. In their landmark paper in 1969, Boyd and Boals reviewed 159 Monteggia-type injuries and noted that the best results were achieved in those patients who underwent rigid internal fixation of the fractured ulna and reduced the radial head. However, when associated with radial head fractures, clinical results have been lower. The best management of Monteggia fracture-dislocations with associated radial head fractures remains controversial due to the literature's lack of consensus.^[9]^

## 2 Case presentation

A 16-year-old right-handed dominant girl presented to the emergency department after a single motorcycle accident. The patient fell and landed on her outstretched left hand. After the crash, she complained of severe pain and deformity of her left forearm. She never had any previous trauma. Her past medical and familial history were not remarkable.

**Figure 3:**
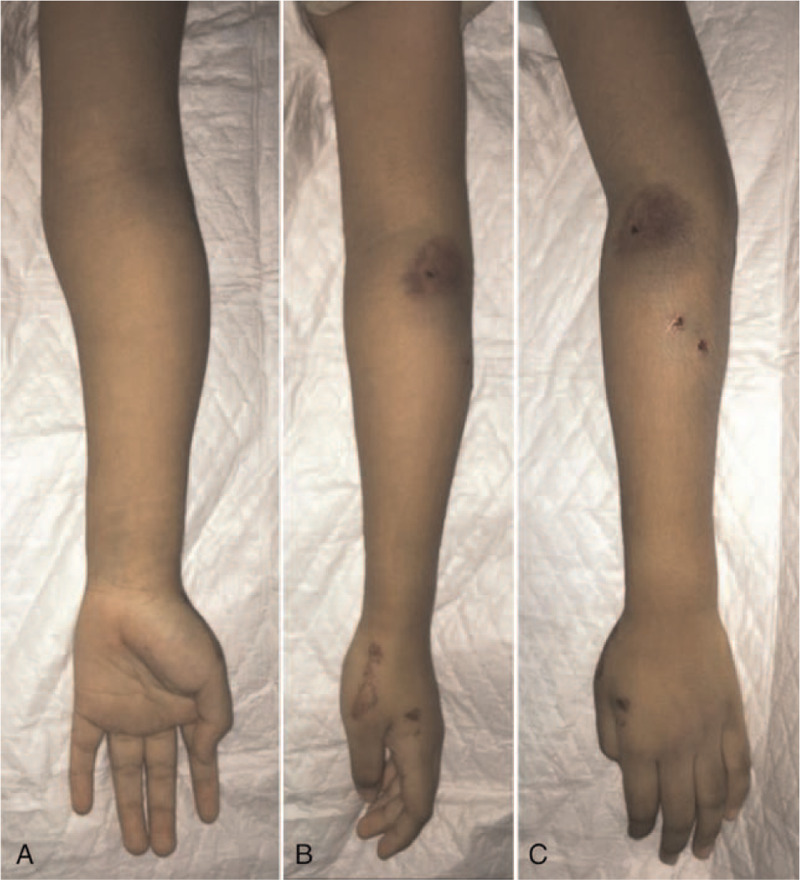
Initial clinical photographs showed deformity and swelling at left forearm (A) anterior view, (B) lateral view, (C) posterior view.

The primary survey revealed a stable hemodynamically. We found an obvious deformity in the left forearm during the physical examination with swelling and limited range of motion due to pain. There was a multiple pinpoint wound at the anterior side of her left elbow and also at the lateral side of her left forearm. The initial clinical photograph is depicted in Figure 3.

**Figure 4:**
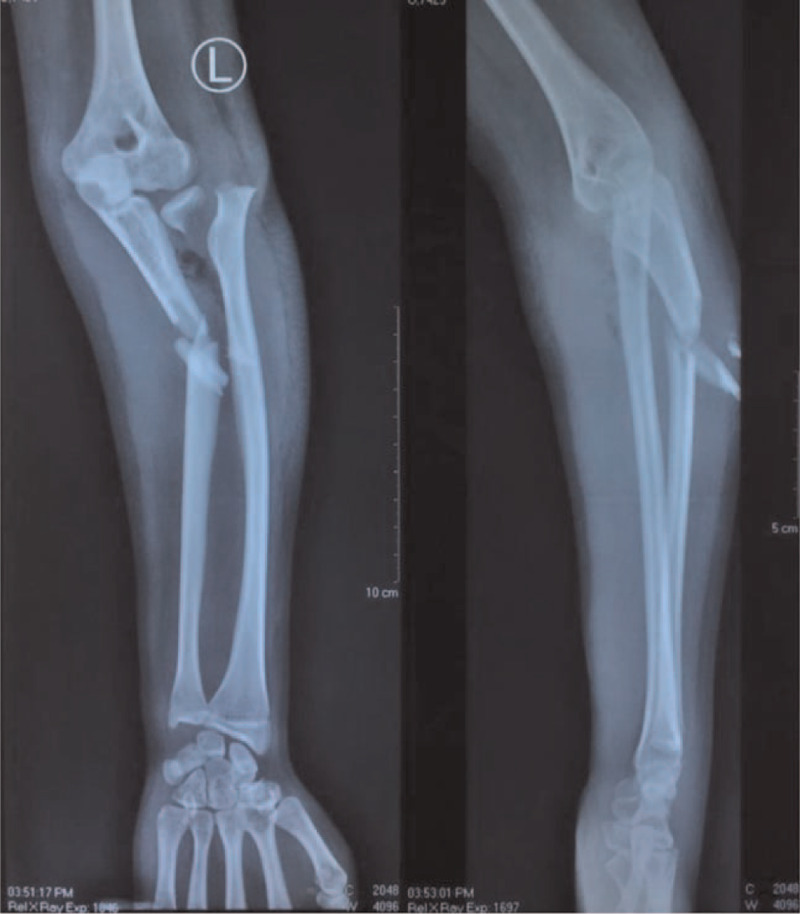
The plain radiograph of the patient's left forearm, anteroposterior (AP) and lateral (L) view.

The plain radiograph examination showed a displaced fracture of the left proximal third ulna, accompanied by a displaced fracture of the proximal radius (Fig. 4).

Based on the physical and radiograph examination, this type of fracture is similar to the Monteggia fracture type III except for proximal radial disruption that occurs laterally through a Salter-Harris type II fracture rather than a radial epiphysis dislocation. In our case, the radial epiphysis remained.

After the accident, the patient immediately underwent surgical debridement. The multiple open wounds were irrigated with large amounts of sterile saline. After the surgical debridement, the forearm was immobilized using a backslap in a supine position and elbow flexion 90^o^.

**Figure 5:**
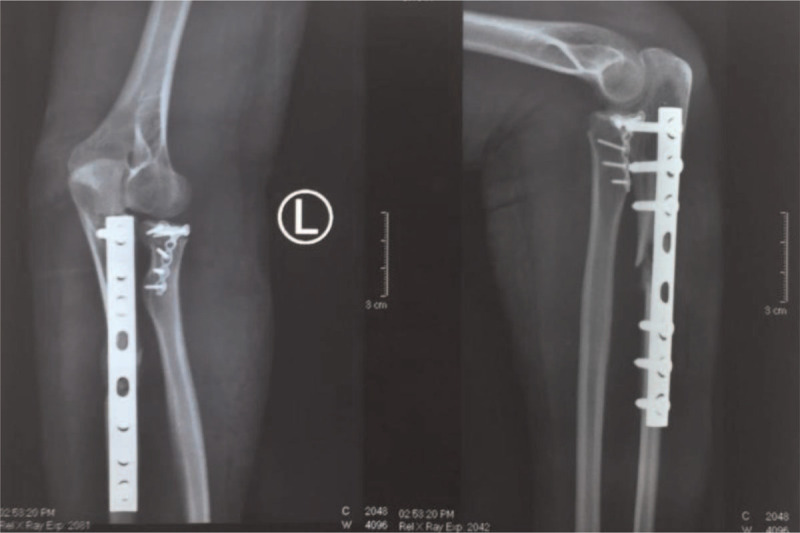
The plain radiograph of the patient's left forearm after open reduction and internal fixation, anteroposterior (AP) and lateral (L) view.

The definitive treatment has been done 5 days later. We performed open reduction and internal fixation of both fractures. The ulna was reduced and stabilized first using a 3.5 mm one-third tubular plate (ORMED). Simultaneous anatomical reduction of the Salter-Harris type II fracture of the proximal radius was achieved, and internal fixation of the radial epiphysis was performed using a 1.6 mm miniplate (Prohealth). The postoperative radiographs are depicted in Figure 5.

**Figure 6:**
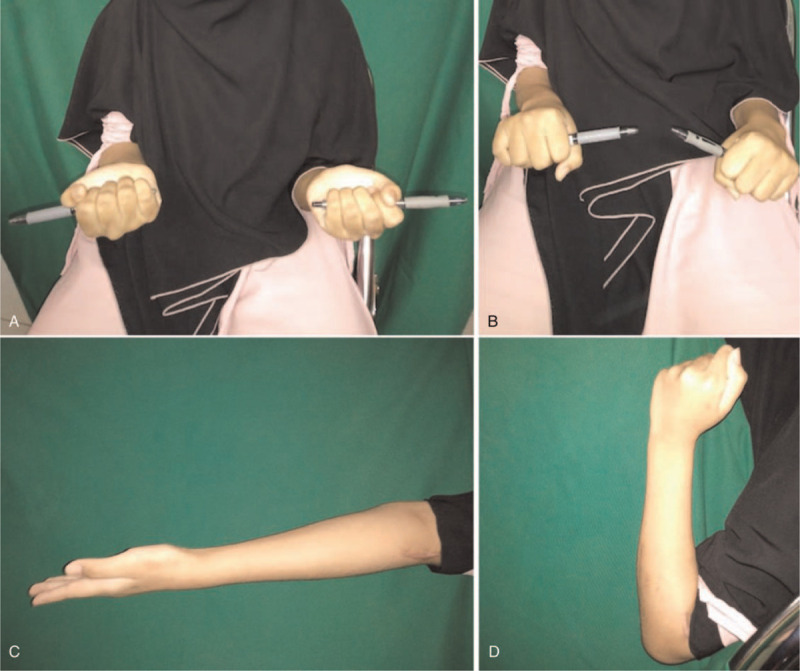
Clinical photographs after 3 months follow-up showed improvement range of motion (ROM) (A) supination position, (B) pronation position, (C) elbow extension, (D) elbow flexion.

Three months after the definitive treatment, the patient's quality of life had improved with the Mayo Elbow Performance Score (MEPS) of 85. She did not complain of any pain, and anatomical reduction of both ulna and radius fractures were well maintained. The patient regained 0 to 125^o^ of elbow flexion and 0 to 165^o^ of supination and pronation (Fig. 6).

## 3 Discussion

Monteggia fracture is a rare injury, and its recommendations of the treatment strategies are sparse. These injuries represent a challenge to orthopedic surgeons. As described by Bado, this fracture consists of dislocation of the proximal epiphysis radius with a fracture of the ulna at various levels. Bado also supplemented his classification to accommodate some unusual varieties, which he called “equivalents” or “Monteggia-like-lesions.”

Neither the previous classifications of Monteggia fracture nor the Monteggia-like-lesion has encompassed the variant described in this case report where a fracture of the ulna shaft combined with a displaced Salter-Harris type fracture of the proximal radial epiphysis. The mechanism of injury is the axial force from the distal to the proximal part of the bone. In this case, the patient was involved with a direct force due to a fall on the outstretched hand.

In his seminal article published in 1967, Bado advocated a nonsurgical approach except for Monteggia type IV fractures and resistant cases. In his report, the nonsurgical approach gave satisfactory results.^[2]^ On the other hand, Boyd preferred the surgical approach by open reduction and internal fixation for acute injuries except for children in whom nonsurgical treatment was employed. In their landmark paper in 1969, Boyd and Boals reviewed 159 Monteggia-type injuries and noted that the best results were achieved in those patients who underwent rigid internal fixation of the fractured ulna and reduction in the radial head,^[10]^ even though the best management of Monteggia fracture-dislocations with associated radial head fractures remains controversial due to the lack of consensus within the literature. Therefore, the surgeon must carefully evaluate the fracture pattern, identify its injury mechanism, and plan appropriate treatment.

Open reduction with internal fixation of the proximal ulnar fracture and radial head is the recognized treatment for Monteggia fracture and Monteggia-like-lesion. The exact anatomic reconstruction of the ulnar alignment (length, rotation, and axis) and stabilization should be the primary goal of surgical treatment to regain unrestricted elbow function.^[11]^ Bugeja et al highly recommend surgical management for type III Monteggia lesions with ipsilateral fracture of the lateral condyles humerus, using percutaneous Kirschner wires to reduce the risk of further displacement and late disability.^[12]^ Locking plates can also be used in Monteggia fracture and Monteggia-like-lesion because of their superior biomechanical stability.^[11]^ The radial head fractures should be treated initially; however, to size the radial head correctly, the ulnar length must be restored by a provisional fixation. The coronoid process is stabilized after the ulnar shaft fracture is treated, and the olecranon is fixed with a dorsal plate.^[13]^

Our case presentation is not a classical dislocation of the radial head; this variant with a Salter-Harris fracture should be considered 1 and must be treated by keeping to the principles of Monteggia fracture reduction. We performed open reduction and internal fixation for both fractures.

At the clinical follow-up examination, MEPS was used to assess the elbow joint's functioning. Three months follow up showed promising results with the MEPS score was 85.

The complexity of Monteggia-like-lesion leads to a variety of outcomes. Children usually show better outcomes than adults. Radial and median nerve injuries are the most common complications that may occur from a rupture or entrapment. This nerve injury incidence was higher in Bado type 2 fractures that cause radial head contusion and compression of the supinator muscle. Treatment of this nerve injury is unnecessary because most patients will recover completely in 9 to 12 weeks. Malunion and nonunion occur in 2% to 10% of cases of Monteggia fractures. This incidence was higher than the average forearm fracture nonunion rate (2%). Other significant complications include acute compartment syndrome (presurgical and postsurgical), radioulnar synostosis (1%–6%), elbow stiffness, heterotopic ossification, ulnohumeral osteoarthritis, and wound infection (0%–3%).^[11]^

The Monteggia-like-lesion described in this report has rarely been previously reported. Our findings demonstrate that good short-term results can be reached if the injury is recognized quickly, classified correctly, and standardized surgical treatment of all injury components is achieved. Further studies with larger patient populations and more extended follow-up periods are needed to evaluate this treatment's long-term effectiveness.

## 4 Conclusion

Monteggia-like-lesions have many variations in physical and radiograph appearance. The diagnosis of this fracture is made through careful evaluation of fracture pattern and identification of injury mechanism. The choice of treatment is selected based on the effectiveness and the principles of Monteggia fracture treatment. Prompt diagnosis and treatment are compulsory to prevent further complications and poor functional results.

## Author contributions

**Conceptualization:** Agung R. B. Santoso, Thomas E. C. J. Huwae, Anindita E. P. Wijaya, Alva Pribadi.

**Data curation**: Agung R. B. Santoso, Thomas E. C. J. Huwae.

**Formal analysis:** Agung R. B. Santoso, Thomas E. C. J. Huwae, Anindita E. P. Wijaya, Alva Pribadi.

**Funding acquisition:** Agung R. B. Santoso.

**Investigation:** Agung R. B. Santoso, Anindita E. P. Wijaya, Alva Pribadi.

**Methodology:** Agung R. B. Santoso, Anindita E. P. Wijaya, Alva Pribadi.

**Project administration:** Agung R. B. Santoso, Thomas E. C. J. Huwae, Anindita E. P. Wijaya.

**Resources:** Agung R. B. Santoso, Thomas E. C. J. Huwae.

**Supervision:** Agung R. B. Santoso.

**Software:** Alva Pribadi.

**Validation:** Agung R. B. Santoso.

**Visualization:** Anindita E. P. Wijaya.

**Writing – original draft:** Agung R. B. Santoso, Anindita E. P. Wijaya, Alva Pribadi.

**Writing – review & editing**: Felix Cendikiawan, Muhammad Abduh, Alva Pribadi, Anindita E. P. Wijaya, Agung R. B. Santoso.
